# Deletion in *MEFV* resulting in the loss of p.M694 residue as the cause of autosomal dominant familial Mediterranean fever in North Western European Caucasians - a case series and genetic exploration

**DOI:** 10.1186/1546-0096-13-S1-O42

**Published:** 2015-09-28

**Authors:** D Rowczenio, D Iancu, H Trojer, J Gilbertson, J Gillmore, A Wechalekar, M Tekman, H Stanescu, R Kleta, T Lane, P Hawkins, H Lachmann

**Affiliations:** 1 National Amyloidosis Centre, University College London, London, UK; 2 Centre for Nephrology, University College London, London, UK

## Introduction

Familial Mediterranean fever (FMF) is the commonest hereditary autoinflammatory disease and it has mostly been reported in populations of Mediterranean ancestry, especially Armenians, Arabs, Turks, non-Ashkenazi and Sephardic Jews. FMF has a very low prevalence amongst Western Europeans. Its hallmarks are autosomal recessive inheritance and short bursts of illness lasting up to 3 days comprising fever, serositis and arthritis, and which can largely be prevented by daily colchicine treatment. The FMF gene *MEFV* is located on chromosome 16p andconsists of 10 exons. Although the disease is usually inherited in an autosomal recessive fashion, deletion of methionine at position 694 has been associated with dominant inheritance.

## Objective

This study characterised the phenotype and response to treatment in patients with p.M694del.

All of the patients were of Northern European heritage, prompting us to performed haplotype reconstruction to investigate the possibility of common ancestry.

## Methods

*MEFV* gene was analysed in 3500 subjects with suspected FMF referred to a single UK centre between 2002 and 2014. Patients with p.M694del underwent additional screening of the *SAA1* gene as well as haplotype reconstruction of the *MEFV* locus.

## Results

The p.M694del variant was identified in 21 patients, sharing an identical disease haplotype that appears to have arisen about 550 years ago. The clinical features comprised typical FMF symptoms with median age at onset of 18 years; three patients presented with AA amyloidosis, of whom two had had symptoms of FMF in retrospect. Fifteen patients had received colchicine treatment, all with excellent responses. The *SAA1.1* allele was found in four patients, including two with AA amyloidosis.

## Conclusion

The p.M694del variant is associated with autosomal dominantly inherited FMF in Northern European Caucasians. Symptoms may develop later in life than in classical recessive FMF but are otherwise similar as is the response to colchicine treatment. The 14% incidence of AA amyloidosis may reflect delay in diagnosis associated with extreme rarity of FMF in this population. The common haplotype suggests a single founder living in about 1460.

